# The Molecular Mechanisms Employed by the Parasite *Myxobolus bejeranoi* (Cnidaria: Myxozoa) from Invasion through Sporulation for Successful Proliferation in Its Fish Host

**DOI:** 10.3390/ijms241612824

**Published:** 2023-08-15

**Authors:** Keren Maor-Landaw, Itamar Avidor, Nadav Rostowsky, Barbara Salti, Margarita Smirnov, Maya Ofek-Lalzar, Liron Levin, Vera Brekhman, Tamar Lotan

**Affiliations:** 1 Marine Biology Department, The Leon H. Charney School of Marine Sciences, University of Haifa, Mt. Carmel, Haifa 3103301, Israel; keren.maor@live.com (K.M.-L.); zangatz@hotmail.com (I.A.); rosnadav1995@gmail.com (N.R.); periparus.ater01@gmail.com (B.S.); vbrekhman@univ.haifa.ac.il (V.B.); 2 Central Fish Health Laboratory, Department of Fisheries and Aquaculture, Ministry of Agriculture and Rural Development, Nir David 1080300, Israel; ritas@moag.gov.il; 3 Bioinformatic Unit, University of Haifa, Mt. Carmel, Haifa 3498838, Israel; maya.lalzar@gmail.com; 4 Bioinformatics Core Facility, llse Katz Institute for Nanoscale Science and Technology, Ben-Gurion University of the Negev, Beer-Sheva 8410501, Israel; levinl@bgu.ac.il

**Keywords:** Myxozoa, parasite, tilapia, calreticulin, histone, sporogenesis, transcriptome, infection

## Abstract

Myxozoa is a unique group of obligate endoparasites in the phylum Cnidaria that can cause emerging diseases in wild and cultured fish populations. Recently, we identified a new myxozoan species, *Myxobolus bejeranoi*, which infects the gills of cultured tilapia while suppressing host immunity. To uncover the molecular mechanisms underlying this successful parasitic strategy, we conducted transcriptomics analysis of *M. bejeranoi* throughout the infection. Our results show that histones, which are essential for accelerated cell division, are highly expressed even one day after invasion. As the infection progressed, conserved parasitic genes that are known to modulate the host immune reaction in different parasitic taxa were upregulated. These genes included energy-related glycolytic enzymes, as well as calreticulin, proteases, and miRNA biogenesis proteins. Interestingly, myxozoan calreticulin formed a distinct phylogenetic clade apart from other cnidarians, suggesting a possible function in parasite pathogenesis. Sporogenesis was in its final stages 20 days post-exposure, as spore-specific markers were highly expressed. Lastly, we provide the first catalog of transcription factors in a Myxozoa species, which is minimized compared to free-living cnidarians and is dominated by homeodomain types. Overall, these molecular insights into myxozoan infection support the concept that parasitic strategies are a result of convergent evolution.

## 1. Introduction

Efficient parasites have the ability to strike a balance between maximal exploitation of host resources and rapid reproduction and transmission. Indeed, parasites are often prolific reproducers that have evolved strategies to evade or suppress the host immune system [1,2]. Myxozoans are a large group of microscopic obligate endoparasites that have recently been placed within the phylum Cnidaria, alongside corals, sea anemones, jellyfish, and hydroids (reviewed by [3]. Myxozoans display highly reduced body plans and genomes, which lack key signaling molecules and transcriptional factors that are hallmarks of multicellularity. However, they retain genes necessary to their function as obligate parasites with complex life cycles [4,5]. The myxozoan life cycle includes two hosts: a vertebrate, mostly fish, and an invertebrate, mostly worms [6,7]. Transmission between hosts is achieved by two distinct types of waterborne spores, termed actinospores and myxospores [8,9].

As with all cnidarians, myxozoans possess complex stinging organelles known as nematocysts, which were previously termed polar capsules. Myxozoan Infection starts with a fast discharge of the nematocyst tubule [10,11], which is triggered by a combination of mechanical and chemical cues [12,13,14]. The launched tubule anchors the spore to the host and allows transmission of the infective sporoplasm. The amoeboid sporoplasm disseminates the infective germ cells into a host species-specific target organ, where sporogony produces the next spore-producing stage [15]. Myxospores are formed in fish within plasmodia, where multicellular proliferative stages occur. The plasmodia undergo intense cell differentiation to generate sporogonic cell stages, which lead to the production of the developed spore [16]. Released myxospores are ingested by worms, where their sporoplasms initiate infection. One infected worm can propagate thousands of actinospores in the water column [17].

Myxozoa is a widespread and large group, currently containing around 2200 species that constitute approximately 20% of all cnidarians [3,16]. Recently, we have identified a new Myxozoa species, *Myxobolus bejeranoi*, as the causative agent of intense infections that have been reported in the last 15 years in Israeli fishponds [18]. *M. bejeranoi* infects the gills of hybrid tilapia at more than 80% prevalence [19] and is capable of infecting fry [20]. Tilapia is the second most cultured fish worldwide [21], and in Israel, it constitutes 60% of total freshwater fish production [22]. Therefore, *M. bejeranoi* infection in hybrid tilapia has a great economic impact on commercial fish farms. Despite the recent development of myxozoan genomic and transcriptomic resources (reviewed by [23]), there are still large gaps in our understanding of the molecular aspects of the infection process.

*M. bejeranoi* is a highly efficient parasite that is capable of rapid proliferation by shutting down the host immune system [19]. Identifying patterns of gene expression in *M. bejeranoi* at the onset of invasion may reveal how this highly compact organism generates thousands of myxospores from an infectious actinospore without much host interference.

To achieve this goal, we conducted a thorough transcriptomic study of *M. bejeranoi* from the initial stages of infecting its hybrid tilapia host through the formation of new spores. Our results show that at the onset of infection, *M. bejeranoi* cells express genes that facilitate rapid cell division, including the required energetic resources. We highlight the potential importance of secreted agents evading host defenses, thereby allowing myxospore development. Overall, these findings shed light on the molecular mechanisms by which *M. bejeranoi* exploits its host for uninterrupted and rapid proliferation and the generation of thousands of new spores.

## 2. Results

**Figure 1:**
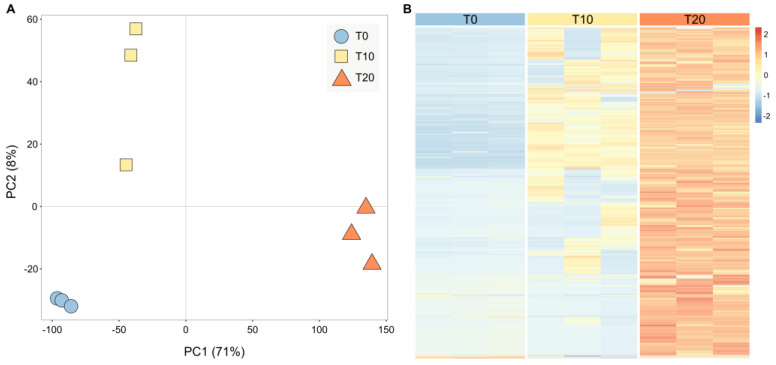
Transcriptome analysis of *M. bejeranoi* at different time points during host infection. (**A**) Principal component analysis (PCA) ordination indicates a time-dependent clustering of samples. (**B**) Heatmap of differentially expressed genes at T0, T10, and T20 after exposure. Rows represent transcripts, and columns represent the tested groups (*n* = 4). Expression level is indicated by the z-score. The full list can be found in Supplementary Table S2.

To identify molecular pathways that are activated in *M. bejeranoi* cells during infection, naïve hybrid tilapia fish were exposed to pond water containing *M. bejeranoi* actinospores for 24 h. Samples were collected immediately (designated as T0), and the remaining fish were transferred to tanks with clean water and sampled again after 10 and 20 days (T10, T20). Parasitic load in fish gills was determined using a previously established technique [19,20], and samples with similar values were selected for transcriptomics (see Section 4). The de novo assembly resulted in 11,399 unique transcripts representing 16,921 transcript isoforms (Table S1). In the current RNASeq study, after mapping and quantification of the reads, we identified the expression of 6098 unique sequences that passed the minimum average count (across samples) of more than one. PCA analysis showed that the biological replicates clustered according to time post-exposure, as T10 samples were transcriptionally closer to T0 than T20 samples (Figure 1A). A similar trend was seen in the hierarchical clustering heat map, where gene expression patterns intensified with the progression of the infection process (Figure 1B). Normalized fold change values showed that most of the differentially expressed genes (DEGs) were upregulated during the 20-day period after initial infection, with 614 DEGs at T10 vs. T0 and 2751 DEGs at T20 vs. T0 (Table S2).

**Figure 2:**
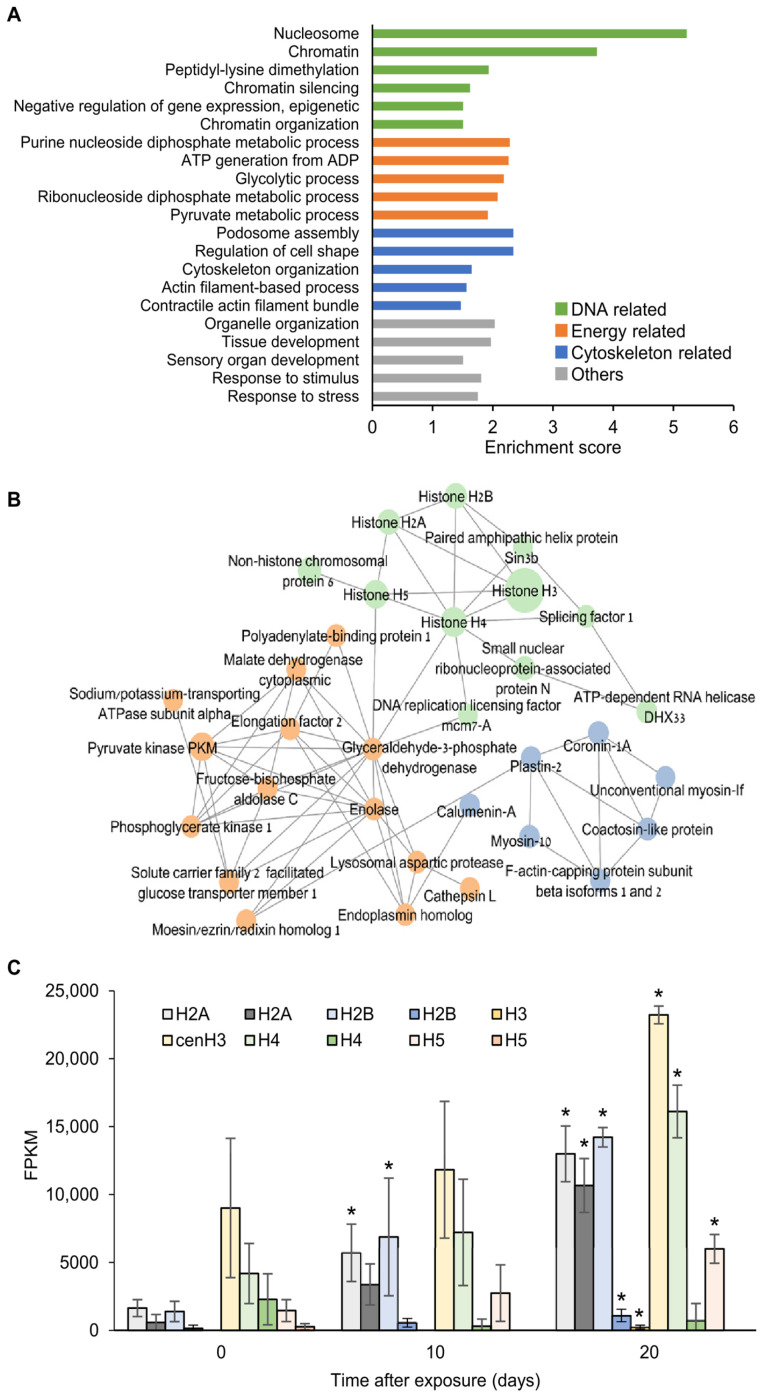
*M. bejeranoi* gene expression at the onset of infection (T0). (**A**) Results of the GO analysis are plotted according to enrichment score. Terms are color-coded for DNA-related, energy-related, actin cytoskeleton-related, and other terms. (**B**) Protein interaction network (STRING database) of T0-expressed genes that were upregulated at T10 or T20. Node size corresponds to T0 expression values, and node colors represent STRING-generated clusters, as in (**A**). (**C**) FPKM values of histone genes at T0, T10, and T20. Asterisks indicate significant differences from T0 (FDR-adjusted *p*-value < 0.05). Details on histone types can be found in Table S4.

To reveal gene expression changes occurring in the myxozoan parasite at the very onset of infection, we compiled a list of 300 genes expressed at T0 (Table S2) and subjected them to GO enrichment analysis (Figure 2A and Table S3). The obtained GO terms were classified into four categories: DNA-related (e.g., nucleosome, chromatin organization); energy-related (e.g., ATP generation from ADP, glycolytic process); actin cytoskeleton-related (e.g., podosome assembly, cytoskeleton organization); and others (e.g., organelle organization, response to stress). Genes expressed at T0 that were later upregulated at either T10 or T20 (a total of 50 genes out of the T0-expressed) were analyzed by generating a protein interaction network using the STRING database with human homologs (Figure 2B). The network, which was significantly enriched with protein–protein interactions (*p* = 3.36 × 10^−10^), was further clustered into three groups using the STRING k-means clustering tool. These gene clusters corresponded to the enriched GO categories, namely, energy-related clusters (e.g., enolase, glyceraldehyde-3-phosphate dehydrogenase); actin cytoskeleton-related clusters (e.g., coactosin, coronin, myosin-10); and DNA-related clusters (e.g., histones, splicing factor 1, ATP dependent RNA helicase).

The list of T0-expressed transcripts included eight histones belonging to six variants [24], including H3, cenH3, canonical H4, canonical H2A, canonical H2B and H5 (Table S4). The expression of most of these histones significantly increased at T10 or T20 (Figure 2C). Among these histones, cenH3 was the most highly expressed throughout the experiment. Two histone transcripts, H3 and canonical H2B, were not expressed at T0 but were upregulated at T20.

**Figure 3:**
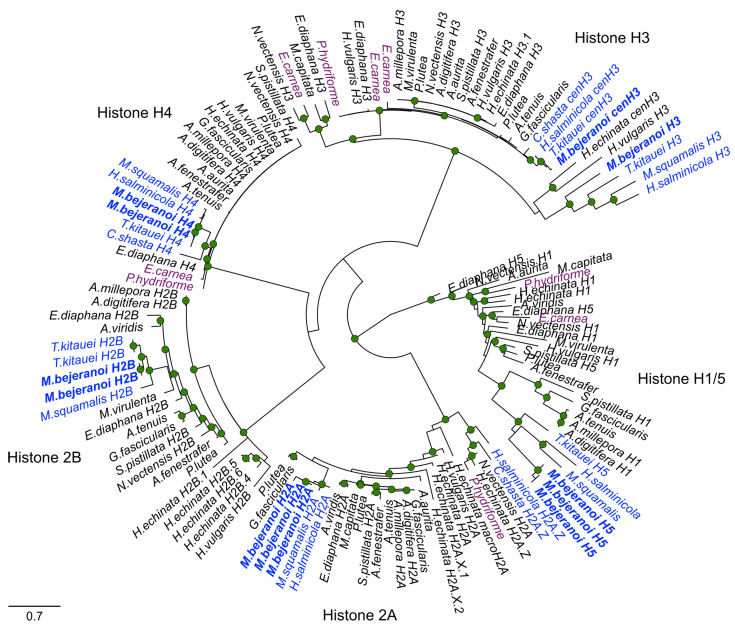
Maximum-likelihood phylogenetic tree of histone gene variants in free-living cnidarians (black), facultative parasitic cnidarians (magenta), and myxozoans (myxosporean-only) (blue). *M. bejeranoi* sequences from this study are in bold. Nodes with bootstrap values higher than 0.7 are shown. See Table S4 for additional information, including NCBI accession numbers.

To generate a histone phylogenetic tree, homologs of variants H1/5, H2A, H2B, H3, and H5 from free-living and parasitic cnidarians were used (Figure 3, Table S4). The HMM classification of the resulting tree placed *M. bejeranoi* histones within their respective clades (Figure 3). However, with the exception of *Ceratonova shasta* H2A and *Henneguya salminicola* H2A, which were classified as H2A.Z, the sequences from Myxozoa formed separate variant clades distinct from those of free-living cnidarians. Myxozoan H3 exhibited two distinct clades: cenH3 and H3.

**Figure 4:**
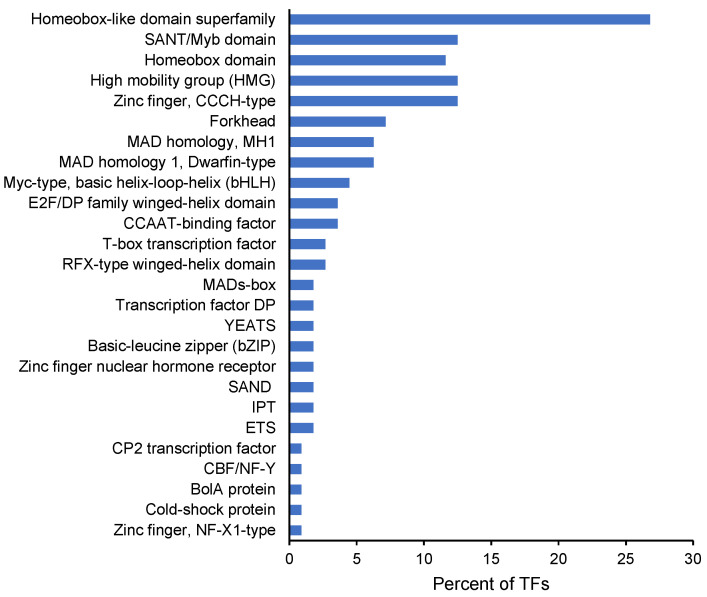
Classification of *Myxobolus bejeranoi* TFs according to their families.

In the list of T0-expressed genes, we identified six transcription factors (TFs) encoding for two forkhead box proteins, a sterol regulatory element, a cyclin-D-binding Myb-like protein, and two high mobility group proteins B1 (HMGB1). One of the latter transcripts, which was upregulated at T20, was shown by the protein interaction network to interact with histone H5 (Figure 2B). To identify additional TFs, we mined the *M. bejeranoi* transcriptome thoroughly, expecting that the genomic and morphological simplicity would be reflected in reduced TF numbers. We found 111 TFs, which were characterized by 26 InterPro domains (Figure 4, Table S5). The most dominant TF type was homeodomain, with 57 domains, followed by zinc finger CCCH-type, with 14 domains. The number of differentially expressed TFs increased from 8 at T10 to 55 at T20 (Table S5).

**Figure 5:**
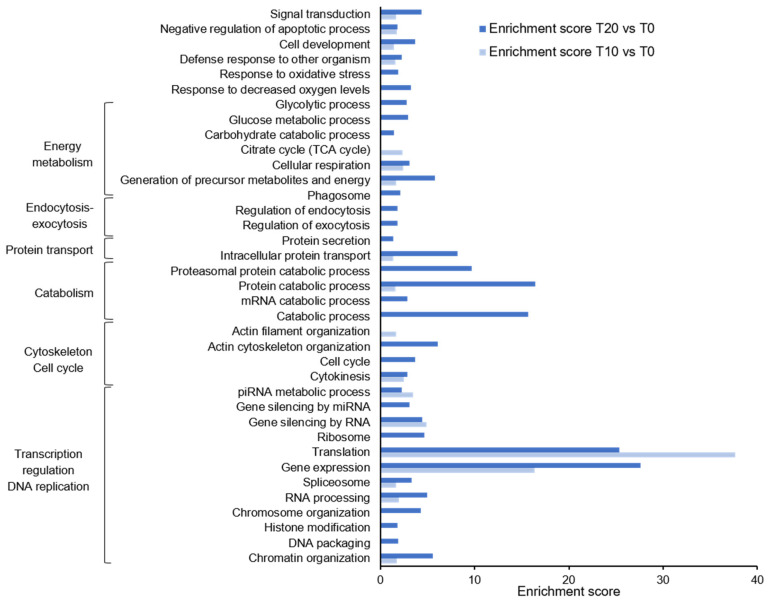
GO enrichment analysis for T10 versus T0 and T20 versus T0. Clusters of cellular processes are marked.

**Figure 6:**
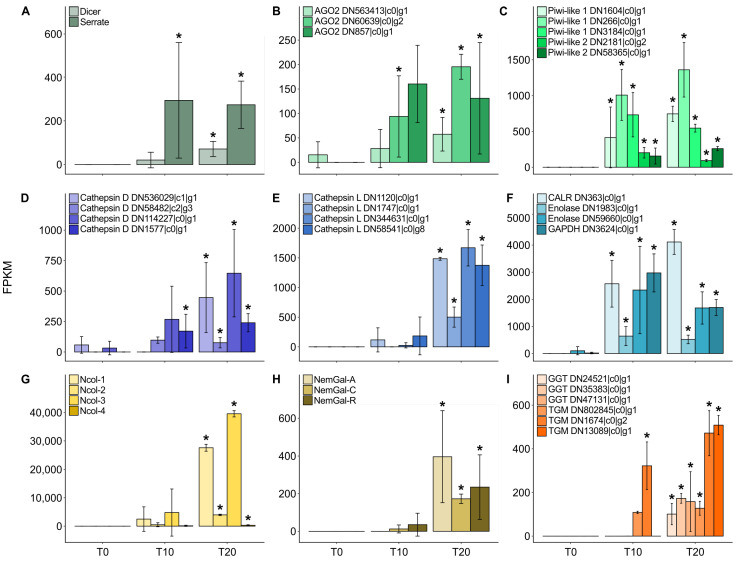
Expression values of genes of interest at 0, 10, and 20 days post-exposure. Mean FPKM values are shown for (**A**,**B**) genes related to RNA silencing by miRNA (DICER, SERRATE, and AGO2); (**C**) genes related to piRNA metabolic processes (Piwi-like proteins 1 and 2); (**D**,**E**) cathepsin proteases; (**F**) calreticulin, enolase, and GAPDH; (**G**) Ncol; (**H**) NemGal; and (**I**) TGM and GGT. Asterisks indicate significant differences from T0 (FDR-adjusted *p*-value < 0.05).

GO enrichment analysis showed that gene expression and translation were the most prominent processes during *M. bejeranoi* infection at both T10 and T20 (Figure 5). Related GO terms, such as RNA processing, chromatin and chromosome organization, spliceosome, and ribosome, were enriched as well. Enrichment of cytokinesis and cell cycle, along with the organization of the actin filament cytoskeleton, was apparent. Processes related to the energetic requirements of the cell (e.g., generation of precursor metabolites and energy, cellular respiration, TCA cycle, glycolytic process) and catabolic processes (protein, RNA) were generally higher at T20. Other terms that were restricted to T20 are response to decreased oxygen levels, regulation of endocytosis, and phagosome. Upregulation of exocytosis regulation 20 days post-exposure was supported by a significant increase in core genes, i.e., synaptotagmin-like protein, syntaxin-binding protein 2, and syntaxin 1B/2/3 [25,26]. Furthermore, gene silencing by microRNA (miRNA) and PIWI-interacting RNA (piRNA) metabolic processes was enriched. The genes governing these cascades, namely endoribonuclease dcr-1 (DICER), serrate RNA effector molecule homolog (SRRT), and three transcripts of protein argonaute-2 (AGO2), which are essential for miRNA gene silencing, and five transcripts of Piwi-like 1 and 2, which are at the core of piRNA processes, were significantly upregulated (Figure 6A–C) [27].

Next, we produced a catalog of *M. bejeranoi* proteases, which included 170 protease transcripts (Table S6) of the types metallopeptidase (35%), cysteine peptidase (20%), threonine peptidase (22%), serine peptidase (12%), aspartic peptidases (7%), and peptidase inhibitors (3%) (Figure S1). The expression of 95 proteases increased with the progression of the infection. Cathepsins, and in particular cathepsin L, were the most highly expressed proteases (Figure 6D,E). Most cathepsins were upregulated, and their predicted localization was extracellular (Table S6). Other metallopeptidases that were predicted to be secreted by *M. bejeranoi* cells are cytosol aminopeptidase and astacin.

**Figure 7:**
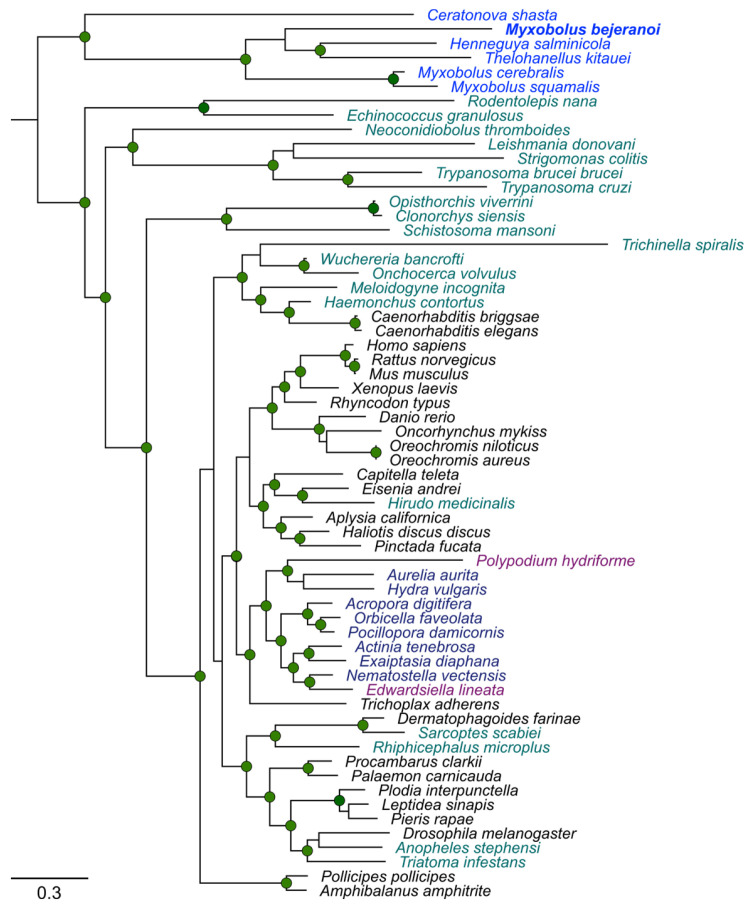
Maximum-likelihood phylogenetic tree of calreticulin homologs in Myxozoa (myxosporean-only) (blue), parasites from other taxa (green), free-living cnidarians (dark blue), facultative parasitic cnidarians (magenta), and non-cnidarian free-living organisms (black). *M. bejeranoi* sequence from this study is in bold. Nodes with bootstrap values higher than 0.7 are shown. Additional information, including NCBI accession numbers, is provided in Table S7.

Interestingly, calreticulin was ranked among the highest differentially expressed genes at both T10 and T20 (Figure 6F and Table S2). Calreticulin has a prominent role in other parasite-host interactions [28]; therefore, we further analyzed myxozoan calreticulin versus those of free-living cnidarians and other parasites. To construct a phylogenetic tree, we assembled 62 calreticulin homologs from various representative taxa (Figure 7, Table S7). Homologs from Myxozoa species *C. shasta*, *M. squamalis*, *M. cerebralis*, *Thelohanellus kitauei*, *H. salminicola*, and *M. bejeranoi* formed a clade that was remotely apart from the clade of free-living Cnidaria, which included representative Anthozoa, Hydrozoa, and Scyphozoa species, and also from parasitic cnidarians with free-living stages, such as Polypodiozoa and the anthozoan *Edwardsiella lineata*.

Lastly, we mined the *M. bejeranoi* transcriptome for taxonomically restricted cnidarian genes that are responsible for nematocyst formation [29]. Minicollagen (Ncol) of types Ncol-1, Ncol-2, Ncol-3, and Ncol-4 were found, as were nematogalectins (NemGal) of types NemGal-A, NemGal-C, and NemGal-related. To validate *M. bejeranoi* types, we generated phylogenetic trees for myxozoan Ncol and NemGal sequences (Figures S2 and S3, Tables S8 and S9). Results showed that *Mb*Ncol and *Mb*NemGal sequences were in close proximity to their respective types in other myxozoans. Interestingly, *Mb*Ncol transcripts started accumulating at T10 and reached extremely high expression values by T20, whereas *Mb*NemGal was upregulated only at T20 (Figure 6G,H). Other upregulated proteins that are important for nematocyst discharge machinery were associated with the biosynthesis of the polymer poly-γ-glutamate [30,31], namely, protein-glutamine gamma-glutamyltransferase (TGM) and gamma-glutamyltranspeptidase (GGT), which were also upregulated mostly at T20 (Figure 6I). The high expression of nematocyst-related genes indicates the beginning of spore formation even at T20.

## 3. Discussion

Although myxozoan parasites affect wild and cultured fish populations worldwide and can cause emerging diseases [32], the molecular mechanisms underlying infection and replication have been understudied as compared to other groups of parasites [33]. Recently, we have demonstrated that *M. bejeranoi* exhibits remarkably efficient suppression of the host immune system [19], which is a common strategy employed by different parasites to evade detection and, thereby, replicate successfully [34,35]. Here, our aim was to uncover the molecular processes that enable *M. bejeranoi* to effectively infect its host in the very early stages of infection.

As in other organisms, developmental processes in Myxozoa involve cell division and the replication of genetic material. Cell division is directly dependent on morphological changes in the actin cytoskeleton, and actin is a prominent regulator of this process [36]. This is consistent with the GO analysis of upregulation of the cell cycle as infection progresses. Additionally, actin cytoskeleton remodeling was established as the driving force of cell motility during myxozoan invasion into the host and during sporogenesis [37,38]. Parasite virulence was strongly linked to migration capacity and the expression of related genes, such as coactosin, coronin, and myosin-10 [38], which are expressed by *M. bejeranoi* at the beginning of infection. The task of replication requires energetic resources, which could be provided by glycolysis, as suggested by the upregulation of glycolytic genes at T0. Indeed, glycolysis is often the main source for ATP production in parasites [39,40].

Our results show that several variants of histones were expressed in *M. bejeranoi* at the onset of infection. Histones are the main genes involved in chromatin organization during cell division. Histone proteins are essential for the packing and protection of the bulk of genomic DNA, but also have important regulatory roles in DNA replication and transcription [41]. In higher eukaryotes, histone gene expression is tightly coupled with DNA replication during the S-phase of the cell cycle, with up to a 20-fold increase compared to non-S-phase levels [42,43]. In cnidarians, histone genes were first characterized in the coral *Acropora formosa* [44]; however, subsequent extensive work in *Hydractinia echinate* [45] and *Hydra magnipapillata* [46] has identified the complete repertoire of histone coding genes. Nevertheless, the presence of histones in myxozoans has been sparsely documented [47,48,49]. Differential expression of histones seems to be a feature of parasitic invasions. Increased expression at the transitional stages leading to a replicating parasite was found to be linked with parasite proliferation in *Plasmodium falciparum* [50], *Leishmania infantum* [51], *Trypanosoma cruzi* [51,52], and the endoparasitic platyhelminth *Mesocestoides corti* [53]. Histones in myxozoan parasites might play a prominent role in their accelerated replication, which commences as early as one day after host invasion.

Parasite-host interactions may include the transfer of materials between the two counterparts. Transfer from host to parasite via endocytosis provides the myxozoan with necessary nutrients [5,54], whereas vesicles exocytosed from parasite to host have been proposed to contain proteins involved in metabolic adaptation to the host environment, tissue invasion, and modulation of the host immune response [55,56]. Based on our findings, we hypothesize that *M. bejeranoi* exosomal vesicles might serve as a means of communication with and influencing the host through the secretion of various proteases, calreticulin, glycolytic enzymes such as enolase and GAPDH, or, alternatively, miRNA gene silencing elements, as has been reported in other parasites.

Recent studies have characterized the vast proteolytic arsenal in myxozoans such as *T. kitauei* [5], *Sphaerospora molnari* [57], *C. shasta* [58], and *Tetracapsuloides bryosalmonae* [54,59]. These are potentially used for parasite migration through tissues, host exploitation, and immune evasion. Proteases of the subfamily cathepsins have received special attention [60]. In particular, cathepsin L and cathepsin D were reported to be highly expressed in sporogonic stages and were suggested to assist in degrading the host tissues and the extracellular matrix, evading the host immune response, and preventing an acute inflammatory response [57,58]. This is consistent with the multiple cathepsin D transcripts we found in *M. bejeranoi*, some of which were expressed at the initial stages of proliferation, and with the extremely high expression of L cathepsin transcripts, which are predicted to be secreted out of the cell [61]. It is thus reasonable to propose that *M. bejeranoi* cathepsins play an important role in its effectivity against the host defenses.

One of the key functional proteins that is highly conserved across different groups of parasites is calreticulin [62,63,64]. This endoplasmic reticulum calcium-binding protein is highly pleiotropic, having many cellular functions in and out of the ER lumen. Its roles in vertebrates include chaperoning, calcium storage and signaling, regulation of gene expression, and cell adhesion (reviewed by [65,66]). Calreticulin was suggested to be a key molecule in host–parasite interaction. In *T. cruzi*, helminths, and arthropod parasites, calreticulin interferes with C1q binding and complement system activation, and modulates the host cellular or humoral immune response (reviewed by [28,67]). Calreticulin is likely to play a prominent role in myxozoan virulence, as it does in other parasites [68]. The discovery that the calreticulin sequences of obligatory parasitic myxozoans form a distinct clade from those of other free-living and facultative parasitic cnidarians suggests that they may serve another function in myxozoans, possibly related to their pathogenesis.

Accumulating evidence suggests that glycolytic enzymes of pathogens, such as parasites of groups Apicomplexa, Leishmania, helminths, and Trypanosomatida, can also be presented extracellularly, either on the plasma membrane or secreted via exosomal vesicles [69,70]. These highly conserved proteins perform multiple functions unrelated to their catalytic activity in glycolysis [69]. Parasitic enolase was suggested to participate in the tissue invasion process and suppress the immune system of the host [69,70,71], whereas GAPDH is thought to modulate the host immune response and inactivate complement C3 [70]. The findings of upregulation of key parasitic proteases, calreticulin, and glycolytic enzymes in *M. bejeranoi* imply the existence of an evolutionarily conserved mechanism associated with pathogenesis that may also be found in myxozoan parasites, both myxosporeans and malacosporeans, and may be a valuable resource for future therapeutic targets [54,59].

Our results showed enrichment of several GO categories related to regulation of gene silencing by miRNA, some even at T10. Cnidarian miRNA encoding and biogenesis are comparable to those of other bilaterians [27,72]. The pre-miRNA molecule is cleaved by ribonuclease Dicer and, along with serrate, forms the miRNA duplex. This duplex further interacts with Argonaute proteins (AGOs) to generate the RNA-induced silencing complex (RISC), which guides the cleavage or translation inhibition of target genes [27]. These three key proteins, which govern the biogenesis of miRNA, were upregulated in *M. bejeranoi* 10 or 20 days post-infection. In mammals, insects, nematodes, and plants, miRNA functions are related to cell differentiation and proliferation, developmental processes, and apoptosis regulation [73]. Other roles in promoting genome stability and regulation of multiple cellular processes were reported in Apicomplexan parasites [74] and protozoan parasites [75]. In organisms such as parasitic flatworms, whose life cycles involve several developmental stages, miRNA has been proposed to play a crucial role in regulating gene expression as a master switch that ensures proper transitions [76,77,78]. Plant pathogenic fungi were enriched with small RNAs at a developmental stage that is characterized by reduced gene expression and metabolism [79]. Myxozoan spores exhibit metabolic dormancy, likely resulting in low levels of total mRNA in mature spores [80]. In the case of *M. bejeranoi*, the core of miRNA biogenesis is upregulated during the later stages of spore development. However, whether miRNAs regulate these dormant stages remains an open question that requires further investigation.

Another possible role for miRNA molecules in parasites is as agents against their hosts. In particular, evidence from plant fungal pathogens [81,82], parasitic nematodes [83,84,85], and parasitic platyhelminthes [86] suggests that miRNA may be designed to silence host immunity genes. miRNAs were reported to be secreted via clathrin-dependent vesicles, which also contained argonaute protein [83]. Our data showed an increase in exocytosis and upregulation of clathrin, which is consistent with the intriguing concept that *M. bejeranoi* inactivates the fish immune system using secreted exosomes that contain gene silencing agents.

A different type of small RNA molecule that we found to be upregulated is piRNA. Their pathway is governed by P-element-induced wimpy testis (PIWI) proteins [87], of which several transcripts were highly upregulated in our study. The PIWI-piRNA system was shown to be essential for the survival of germlines and for silencing transposable elements in mice and Drosophila [88]. Additionally, the protozoan parasite T. cruzi secreted tRNA-derived small RNAs and PIWI proteins in clathrin-dependent vesicles into the extracellular medium and susceptible host cells [89]. The idea that *M. bejeranoi* utilizes sRNAs to suppress its host defense systems or to regulate the spore resting state is most compelling. Further investigation in this direction may yield important insight into Myxozoa-host interplay and the molecular regulation of myxozoan life cycle transitions.

Cnidarian TFs are comparable to those of bilaterians [90,91]. However, TF diversity is expected to correlate with morphological and cell type complexity [90,92]. Indeed, the *M. bejeranoi* TF repertoire contained three times fewer TFs than the cnidarian moon jellyfish *Aurelia aurita* [93]. We found that the homeodomain, which is a hallmark of development and cell differentiation processes in metazoans [94], is the dominant type of TF in the *M. bejeranoi* repertoire. Several transcripts encoding homeobox proteins were previously detected in a two-host transcriptome analysis of the myxozoan *Tetracapsuloides bryosalmonae* [59], implying their importance in myxozoan development. The second dominant type in the *M. bejeranoi* repertoire was the high-mobility group (HMG), which is involved in chromatin remodeling and cell fate determination [95]. Interestingly, the C2H2 zinc finger, which is the most common in the moon jellyfish and in vertebrates [93,96], was missing from the parasite repertoire. However, another zinc finger type, the CCCH type, was relatively abundant in *M. bejeranoi*. Another example is the transcription factor p53, which has an important role in controlling the cell cycle and programmed cell death. Along with other core apoptotic proteins, p53 was shown to be diminished in the transition from free-living to parasitic cnidarians [97]. However, because our sample did not represent all stages of the myxozoan life cycle, we cannot conclude with certainty that these TF family types are missing from the *M. bejeranoi* transcriptome. Most of the TFs were highly expressed during sporogenesis, where cell division and differentiation take place. Nevertheless, during sporogenesis, the myxozoan cells differentiate into only three types of cells, nematocytes, valves, and sporoplasms, lacking other common cell types such as neurons, muscles, and cilia. Therefore, it will be interesting to address the role of TFs that function in processes such as neuronal development, for example, Fox and Sox [98,99,100,101]. This is the first report of the transcription factor repertoire in a Myxozoa species, and further studies might answer important questions regarding the regulation of these processes by such a compact genome [4].

Plasmodium endogenous cell differentiation and proliferation produce a new stage of myxospores. Towards the end of sporogenesis, at the spore-forming stages, the myxozoan nematocyst is generated [16]. Myxozoans harbor the two cnidarian-restricted gene families of minicollagen and nematogalectin, whose products are the main building blocks of the nematocyst structure [29]. The expression of minicollagens was restricted to those stages and was negligible during presporogonic development [102]. Additionally, biosynthesis of the polymer poly-γ-glutamate, which drives the osmotic machinery that enables nematocyst discharge, occurs late in the capsule differentiation process [30,31]. Our analysis showed enzymes associated with poly-γ-glutamate biogenesis, as well as four types of minicollagen and three types of nematogalectin, to be differentially expressed mostly at T20. These results enable us to pinpoint the general timeline of *M. bejeranoi* sporogenesis, suggesting that by day 20 post-infection, the newly generated spores are in the final developmental stages.

**Figure 8:**
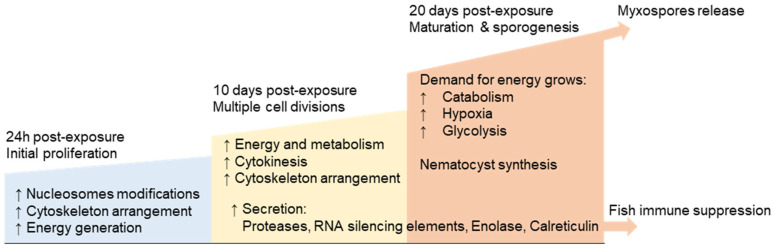
A model illustrating the key findings on the cellular processes occurring in *M. bejeranoi* while infecting its hybrid tilapia host. At the onset of infection (24 h post-exposure), nucleosome modifications governed by the expression of histone genes, cytoskeleton alterations, and energetic resources are essential for commencing accelerated proliferation. After 10 days, multiple cell divisions take place, demanding high energy. At the same stage and during the next 10 days, the secretion of proteases and possibly RNA silencing elements, enolase, and calreticulin probably modifies the surrounding gill tissue and the fish immune system. As sporogenesis progresses, the number of oxygen-consuming cells increases, leading to hypoxia. Additionally, the growing demand for energetic resources may be supplied by catabolic processes and glycolysis. Nematocyst synthesis occurs at the final stages of spore maturation, just before fully developed myxospores are released into the water column, ready to infect the next host.

Overall, our transcriptomic data illuminate the molecular basis of *M. bejeranoi* infection and provide the first transcription factor repertoire of a myxozoan species. Our findings highlight important molecular players that could be involved in its pathogeny, fast proliferation, and suppression of the host immune system, while providing a basic timeline for sporogenesis (Figure 8). Parasitism has evolved independently multiple times in various taxonomic groups [100,103]. As all parasites face similar challenges related to transmission between hosts, invading and surviving within the host, and utilizing its resources effectively for reproduction, natural selection has pushed these unrelated lineages into shared paths [100]. Our findings suggest that this theory of convergent evolution is also valid in the case of a basal myxozoan parasite such as *M. bejeranoi*.

## 4. Materials and Methods

### 4.1. Fish Infection Experimental Design

On 22 August 2021, 300 naïve hybrid tilapia fish with a mean weight of 2.98 g that had hatched on 30 June 2021 were introduced to the pond using three confined cages of ~100 L (100 cm × 30 cm × 30 cm). The mean water temperature, which was recorded constantly during the experiment using a temperature data logger (HOBO), was 30.25 °C. Before the experiment, five representative fish were subjected to a thorough parasitological examination, which was negative. Additionally, quantitative PCR (qPCR) ruled out low levels of *M. bejeranoi* DNA in the gills of the naïve fish (n = 41) [20]. After a 24 h exposure to the pond water, the fish were randomly translocated to three 100-L indoor tanks at the Central Fish Health Laboratory, Nir David. Tanks had a flow-through system with dechlorinated tap water at a temperature of ~25 °C. The fish were fed daily with commercial fish pellets.

Fish were sampled (n = 20–30) immediately and at 10 and 20 days post-exposure (time points (T) 0, T10, and T20, respectively). At each time point, fish were euthanized using 1 mL/L 2-phenoxyethanol and whole-gill tissue (four and a half gill lamellae) from one side was collected and snap-frozen in liquid nitrogen. To avoid tank effects, each sampling included fish from all three tanks.

### 4.2. RNA and DNA Extraction and Evaluation of Infection Severity

RNA and DNA were extracted from gill tissue simultaneously, as previously described [19]. Briefly, tissue was lysed in TRIzol Reagent (Thermo Scientific, Waltham, MA, USA), and DNA and RNA phases were separated using chloroform. RNA was treated with DNAse I (Ambion, Austin, TX, USA) according to the RNA Clean & Concentrator-25 kit protocol (Zymo Research, Irvine, CA, USA). The concentration of RNA and DNA was measured using a NanoDrop 2000c spectrophotometer (Thermo Scientific), and RNA integrity (RIN > 7.5; mean, 8.5) was assessed by a 2200 TapeStation System (Agilent Technologies, Santa Clara, CA, USA). In addition, we manually isolated 25 cysts from infected gills and extracted RNA using a Zymo RNA Clean & Concentrator TM-5 kit (Zymo Research) method (RIN = 7.1).

The infection severity of *M. bejeranoi* in fish gills was evaluated by qPCR, as previously described [19]. Briefly, specific primers targeted to amplify the *M. bejeranoi* small subunit ribosomal RNA gene (SSU rDNA), along with primers for Tilapia β-actin as a normalizer, were used on the extracted DNA. The computed qPCR relative quantity (RQ) was denoted as the relative infection severity index. From each sampling time point, three RNA replicates with similar mean RQ values (T0, 1.35 ± 0.21; T10, 7.65 ± 2.61; T20, 54 ± 8.84) were sent for sequencing.

### 4.3. Sequencing and Transcriptome Assembly

For RNA-seq, library preparation and sequencing were conducted by the Technion Genomics Center, Haifa, Israel. Twelve RNA-seq libraries (3 non-infected gills and 9 infected gills) were prepared from purified mRNA and constructed simultaneously using the NEBNext Ultra II Directional RNA Library Prep Kit for Illumina, according to the manufacturer’s protocol. The RNA-seq data were generated on Illumina NextSeq2000 using P3 300 cycles (2 × 150 paired-end). To supplement myxozoan sequencing data, we used three additional samples of infected gills and one sample of *M. bejeranoi* cyst that was manually excised in August 2020, which were sequenced in BGI on the BGISEQ-500RS platform using 100-bp paired-end reads. These four raw reads were filtered by SOAPnuke (version 2.1.0) using default parameters to remove low-quality reads. Then, de novo assembly was done without additional filtration using Trinity (version 2.9.0.) with default parameters (named de novo assembly A). The same reads were de novo assembled again after mapping to the Oreochromis aureus genome (ASM587006v1) using the STAR aligner v.2.5.2b (de novo assembly B). The raw reads of the 12 experimental samples and 4 supplementary samples were analyzed together using the NeatSeq-Flow platform [104]. The sequences were quality trimmed and filtered using Trim Galore (version 0.4.5) and cutadapt (version 1.15).

To assemble a host-free parasite transcriptome, the filtered reads were first mapped to the host genomes (O. niloticus, GCF_001858045.2, and O. aureus, ASM587006v1) using the BWA program [105] (version 0.7.17 with mod mem). Unmapped reads identified as rRNA by the SortMeRNA program [106] were further removed. The remaining reads were further taxonomically classified by using Kaiju [107], and reads classified as Bacteria, bony fish, or Hominoidea were removed for downstream analysis. Then, the lasting reads were assembled into transcripts using Trinity [108] (Version 2.8.4) (de novo assembly C). A similar protocol was repeated without the taxonomic filtration (de novo assembly D).

To obtain contamination-free transcriptomes from assemblies A-D, transcripts were BlastX-searched against the NCBI non-redundant database (download 20 September 2022) and only transcripts with a hit to Cnidaria (E-value below 1 × 10^−5^ and minimum coverage of 50%) were further analyzed. To generate a representative transcriptome containing only unique transcripts from the resulting transcripts of the four assemblies, CD-HIT [109] was used with a sequence identity cut-off of 98%.

The resulting non-redundant dataset contained 16,921 transcripts (Table S1), of which 2713 were derived from assembly A, 7420 from assembly B, 4781 from assembly C, and 2007 from assembly D. The dataset was screened for assembly quality and completeness by identifying BUSCO genes (http://busco.ezlab.org/) (accessed on 12 December 2022) using the Eukaryota BUSCO v5 dataset [110]. The results (63.53%; complete 50.59% and fragmented 12.94%) are in line with other studies of myxozoans [54], demonstrating that BUSCO underestimates completeness in organisms with reduced genomes.

Reads were then mapped to the representative transcripts (Table S10) using RSEM [111] (version 1.3.1) and Bowtie2 [112] (version 2.3.5.1) and gene-level annotation was done using the Trinotate pipeline [113].

### 4.4. Differential Gene Expression Analysis and Annotation

To identify differentially expressed genes, statistical testing, normalization, clustering, and enrichment analysis were performed using the DESeq2 module in the NeatSeq-Flow platform [104]. RLOG normalization was used for a visual representation of the results as well as for clustering analysis. Clustering was done using the hclust R function (metric: Pearson and method: ward.D2), and the number of clusters was determined by the eclust R function. Statistical analysis was done using the DESeq2 [114] R package. The statistical model considered one effect for comparison (Contrast) between the different “Time” states. The likelihood ratio test was also used to determine the statistical contribution of the “Time” states. The analyses produced a *p*-value, an FDR-adjusted *p*-value, and a fold change per gene. Genes with an FDR-adjusted *p*-value < 0.05 were considered differentially expressed. Quality was assessed using FASTQC (version 0.11.8), MultiQC [115] (version 1.0.dev0), and Quast [116] (version 5.0.2).

PCA was performed using the DESeq2 R package plotPCA function code, and heatmaps were generated using the pheatmap R package. Annotation was done using Trinotate against Swissprot (1/2021). A BlastX search of transcripts against the *Homo sapiens* dataset was performed using the Galaxy interface (01/2023) [117], with default parameters. *H. sapiens* homologs of the Kyoto Encyclopedia of Genes and Genomes (KEGG) and Gene Ontology (GO) enrichment analyses were performed using the clusterProfiler [118] (v3.16.0) R package. For T0 GO enrichment, a list of T0-expressed genes was generated using a cumulative normalized expression value greater than 5 with no more than one biological sample with 0 expression in any replicate. Human homologs of T0-expressed genes were used to create a network of interconnected proteins using the STRING 11.5 database [119]. Protein networks of T0-expressed genes that were upregulated at T10 or T20 were exported to Cytoscape [120] for graphical editing. Transcripts of histone genes were classified according to their variants using HMM-profile scores in HistoneDB 2.0 [24]. Histone homologs from other myxozoans were mined and assessed using the HistoneDB tool as well. Transcription factors (TFs) were identified by Interpro according to their family groups, as described before [96].

The *M. bejeranoi* protease catalog was produced by inputting KO numbers into the KEGG Mapper—Reconstruct tool [121,122] and using Brite to map predicted peptidases and inhibitors in the transcriptome (Ko01002). The subcellular localization of differentially expressed proteases was predicted bioinformatically using DeepLoc version 2.0 [61].

### 4.5. Phylogenetic Trees

Sequences were obtained from NCBI and from the reef genomics website (http://reefgenomics.org/) (accessed on 3 April 2023) [123] using blastp (E-value 1 × 10^−5^ and minimum coverage of 30%, 03/2023). Protein alignment was conducted using MAFFT v7 [124]. Maximum likelihood trees were generated in PhyML v3.0 [125] and visualized using FigTree v1.4.4 (http://tree.bio.ed.ac.u/ accessed on 3 April 2023)).

## Data Availability

The data supporting the findings of this study are presented in the main text and its additional files. The raw sequence data were deposited in the NCBI SRA database under Bioproject accession PRJNA995317. This Transcriptome Shotgun Assembly project was deposited at DDBJ/EMBL/GenBank under the accession GKNV00000000. The version described in this paper is the first version, GKNV01000000. *M. bejeranoi* histones accession numbers OR427312-22; calreticulin OR413615, minicollagens OR413616- OR413619 and nematogalectins OR413620- OR413622.

## Supplementary Materials

The following supporting information can be downloaded at: https://www.mdpi.com/article/10.3390/ijms241612824/s1.
