# Supplementary material for: The Molecular Mechanisms Employed by the Parasite Myxobolus bejeranoi (Cnidaria: Myxozoa) from Invasion through Sporulation for Successful Proliferation in Its Fish Host
Source: Int J Mol Sci. 2023 Aug 15;24(16):12824. doi: 10.3390/ijms241612824 (PMC10454682; doi:10.3390/ijms241612824)
Supplement: Supplementary file 1 [file ijms-24-12824-s001.zip › Supplementary Figures Aug_8_23.pdf]

Supplementary Figures:

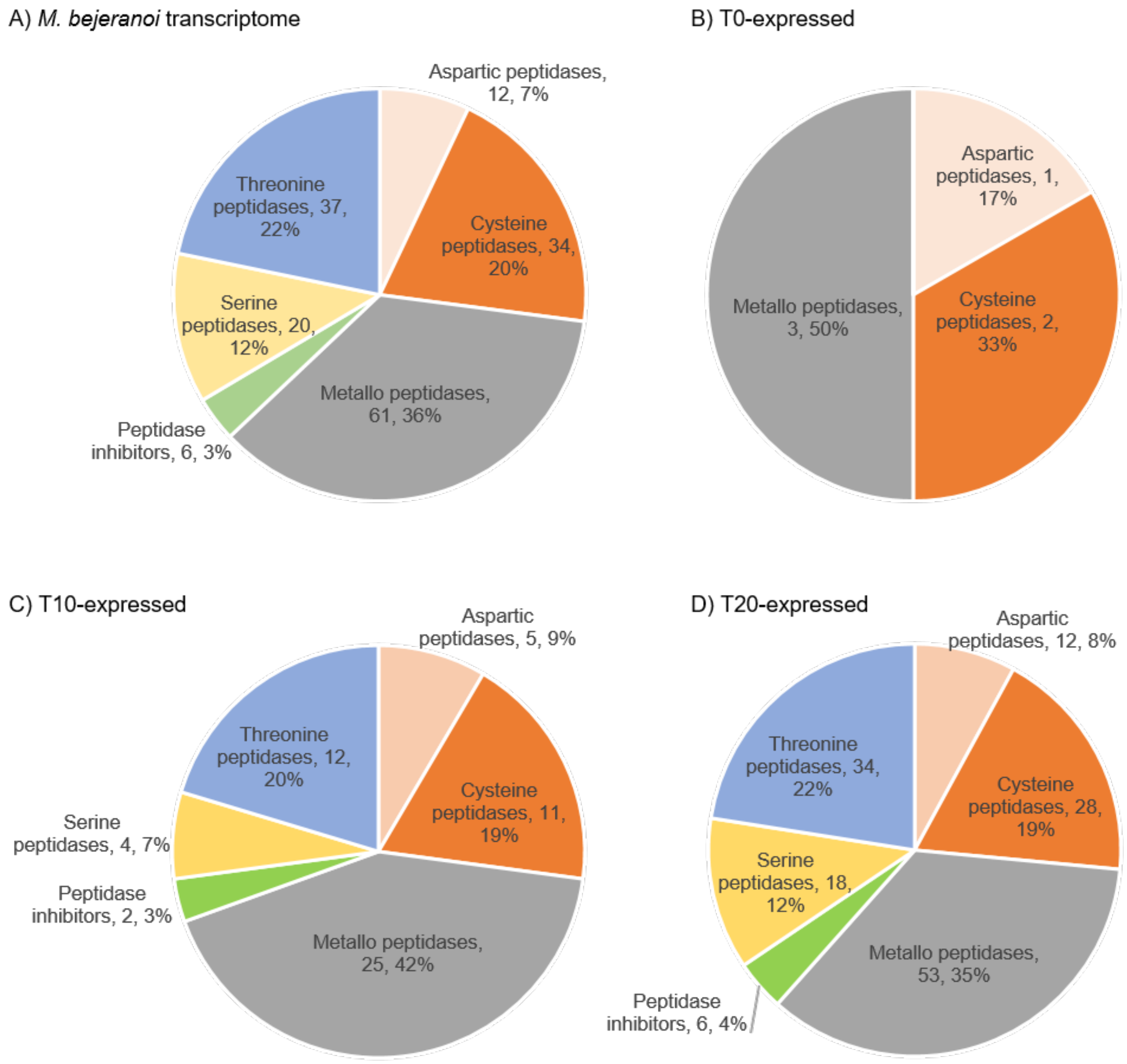

Figure S1. Pie charts showing diversity and abundance of protease types in *M. bejeranoi* transcriptome (A) and their distribution at T0, T10, and T20 (B-D).

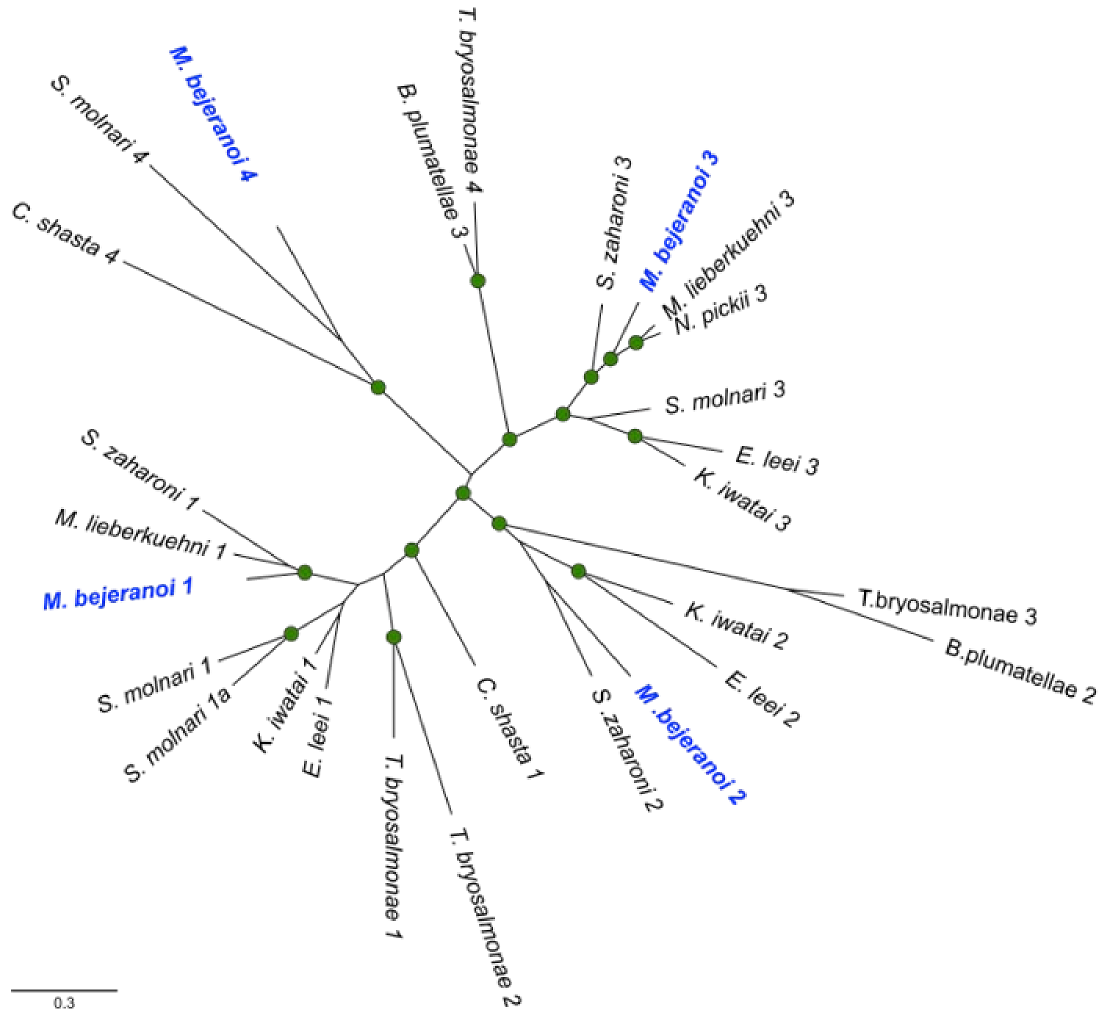

Figure S2. Maximum-likelihood phylogenetic tree of myxozoan Ncol 1-4. Nodes with bootstrap values higher than 0.7 are shown. Additional information, including NCBI accession numbers, is provided in Table S8.

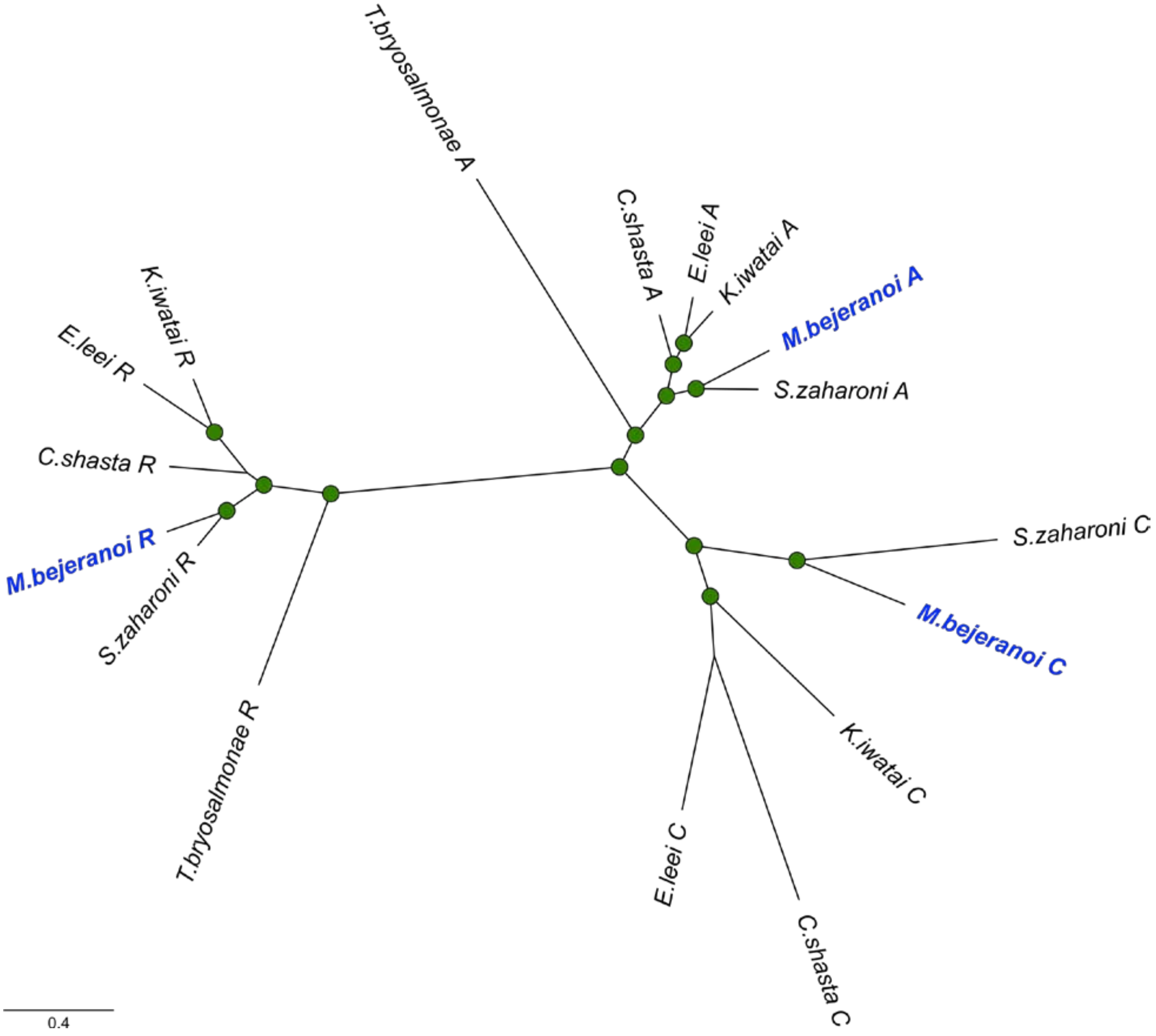

Figure S3. Maximum-likelihood phylogenetic tree of myxozoan NemGal of types A, C, and related (R). Nodes with bootstrap values higher than 0.7 are shown. Additional information, including NCBI accession numbers, is provided in Table S9.
